# A Quantitative Approach to Analyzing Genome Reductive Evolution Using Protein–Protein Interaction Networks: A Case Study of *Mycobacterium leprae*

**DOI:** 10.3389/fgene.2016.00039

**Published:** 2016-03-29

**Authors:** Richard O. Akinola, Gaston K. Mazandu, Nicola J. Mulder

**Affiliations:** ^1^Computational Biology Group, Department of Integrative Biomedical Sciences, Medical School, Institute of Infectious Disease and Molecular Medicine, University of Cape TownCape Town, South Africa; ^2^Department of Mathematics, Faculty of Natural Sciences, University of JosJos, Nigeria; ^3^African Institute for Mathematical SciencesCape Town, South Africa; ^4^African Institute for Mathematical SciencesCape Coast, Ghana

**Keywords:** genome reductive evolution, protein–protein interactions, *Mycobacterium leprae*, functional analysis

## Abstract

The advance in high-throughput sequencing technologies has yielded complete genome sequences of several organisms, including complete bacterial genomes. The growing number of these available sequenced genomes has enabled analyses of their dynamics, as well as the molecular and evolutionary processes which these organisms are under. Comparative genomics of different bacterial genomes have highlighted their genome size and gene content in association with lifestyles and adaptation to various environments and have contributed to enhancing our understanding of the mechanisms of their evolution. Protein–protein functional interactions mediate many essential processes for maintaining the stability of the biological systems under changing environmental conditions. Thus, these interactions play crucial roles in the evolutionary processes of different organisms, especially for obligate intracellular bacteria, proven to generally have reduced genome sizes compared to their nearest free-living relatives. In this study, we used the approach based on the Renormalization Group (RG) analysis technique and the Maximum-Excluded-Mass-Burning (MEMB) model to investigate the evolutionary process of genome reduction in relation to the organization of functional networks of two organisms. Using a *Mycobacterium leprae* (MLP) network in comparison with a *Mycobacterium tuberculosis* (MTB) network as a case study, we show that reductive evolution in MLP was as a result of removal of important proteins from neighbors of corresponding orthologous MTB proteins. While each orthologous MTB protein had an increase in number of interacting partners in most instances, the corresponding MLP protein had lost some of them. This work provides a quantitative model for mapping reductive evolution and protein–protein functional interaction network organization in terms of roles played by different proteins in the network structure.

## 1. Introduction

Worldwide DNA sequencing efforts have led to a rapid increase in sequence data for many organisms in the public domain. Comparative genomics analyses have yielded many valuable insights into genome relatedness and dynamics of organizational complexity of these genomes, including their sizes, gene content and other essential features, such as adaptation to their environment. In the case of bacterial species, for example, a variation in sizes of their genomes has been observed, revealing that intracellular bacteria commonly have a reduced genome size, as a consequence of their nutritional dependence on, and adaptation to their host environment and specialization (Tamames et al., 2007; Gil and Latorre, 2012; Rosinski-Chupin et al., 2013). This results in inactivation or loss of genes within the bacterial genome, resulting in reductive evolution, where several ancestral genes have been rendered non-essential and completely removed from the genome (Tamames et al., 2007; Gil and Latorre, 2012). In the context of mycobacterial species, *Mycobacterium leprae* has the smallest genome as a result of massive reductive evolution, compared to *Mycobacterium tuberculosis*, while both have an increasingly parasitic lifestyle in the host compared to other mycobacteria (Han and Silva, 2014).

The genome sizes of MLP and MTB are 3,268,203 and 4,411,532 base pairs, respectively (Cole et al., 2001). Thus, the genome of MLP is approximately 1.4 Mb smaller than MTB. In addition, the G+C content of MLP is 57.7% which is lower than other mycobacterial genomes, while that of MTB is 65.5%. Although MTB and MLP share a common ancestor, MLP is an obligate intracellular parasite, while MTB is a facultative intracellular parasite (Youm and Saier, 2012). Youm (Youm and Saier, 2012), compared the clinical CDC1551 strain of MTB (4189 proteins) to the TN strain of MLP (1605 proteins) and proposed two main consequences of the reduction in the genome of MLP (Cole, 1998): the presence of few proteins belonging to the PE and PPE functional category and traces belonging to insertion sequences and bacteriophages. As shown in Table S1 in Akinola et al. (2013), the number of proteins in the MTB genome belonging to the PE and PPE family is roughly fifteen times that of MLP, and while 82 proteins in MTB are insertion sequences or derived from bacteriophages there are only two in MLP.

Gómez-Valero et al. (2007) defined reductive evolution as the process by which genes and their corresponding functions are lost, resulting in the downsizing of the genome. Three reasons based on changes in lifestyle were given why an organism may have reductive evolution: a desire to “move” from a free living to a host-associated or intracellular life, when the organism restricts itself from multiple to specific hosts and from multiple to specific host tissues. The presence of pseudogenes in MLP and the corresponding absence thereof in MTB accounts for some of the genotypic differences between the two pathogens with remarkable disease phenotypic differences in their host. MLP infection leads to leprosy, which is a chronic dermatological (Monot et al., 2009) and malignant human neurological disease (Cole et al., 2001), affecting mainly the skin, peripheral nerves, the eyes and mucosa of the upper respiratory tract (World Health Organization, 2012). On the other hand, MTB infection leads to tuberculosis (TB), one of the “most dangerous” infectious diseases, affecting mainly lungs (Mazandu and Mulder, 2012) with active pulmonary tuberculosis.

MLP's highly reduced genome makes it an interesting species as a model for reductive evolution within a genus with ancestral genes classified into three categories (Gómez-Valero et al., 2007), namely retained, absent/deleted and pseudogenized. Genes belonging to the “absent” category have either diverged so much that they cannot be recognized or were totally deleted, while those in the pseudogenized category have sufficient levels of nucleotide similarity with MTB. These pseudogenes that are found in MLP are inactivated versions of genes that are still functional in MTB. These are most likely the remains of genes that have lost their functions, for example, by acquiring nutrients from the host, as constrained by their intracellular lifestyle (Tamames et al., 2007; Rapanoël et al., 2013). It was also reported that 1537 genes have been lost from the ancestor to MLP, of which, 1129 are pseudogenes (Gómez-Valero et al., 2007). Different features related to evolutionary processes were elucidated mostly using comparative genomics analyses, and so far only Tamames et al. (2007) have used the modular organization of protein–protein interaction networks to analyze the reductive evolution in the Buchnera genome compared to the *E. coli* genome.

In this work, we use protein–protein functional interactions generated for both MLP and MTB and ortholog data to study reductive evolution using the Renormalization Group (RG) analysis technique and the Maximum-Excluded-Mass-Burning (MEMB) model. This is based on the premise that both organisms descended from the same ancestral mycobacterium. In a recent study (Akinola et al., 2013), using ortholog data, we found 2859 proteins out of 4136 proteins interacting in the MTB functional network alone and 1277 that are shared between MLP and MTB functional networks. Here, we extend this study to analyze these 2859 proteins unique to MTB and the 1277 shared between them under the transformation of the MTB functional network into successive smaller copies of itself (Gallos et al., 2012) to reveal different biological features that are able to explain reductive evolution in MLP in comparison to its closely related MTB genome.

## 2. Materials and methods

To analyze reductive evolution in the MLP genome compared to the MTB genome, we used previously generated MLP and MTB functional networks (Akinola et al., 2013). These functional networks were obtained by combining protein interaction data from multiple sources, including the STRING database (Jensen et al., 2009; Franceschini et al., 2013), other functional data, such as sequence and microarray data, and protein–protein interaction (PPI) datasets (Salwinski et al., 2004; Yellaboina et al., 2009, 2011; Licata et al., 2012; The UniProt Consortium, 2015). We mapped different protein identifiers from different sources to UniProt Accession numbers using datasets for the two mycobacterial organisms: *Mycobacterium leprae* and *Mycobacterium tuberculosis* downloaded from the UniProt database (The UniProt Consortium, 2015). We applied the Renormalization Group (RG) analysis technique (Song et al., 2005; Gallos et al., 2008, 2007a,b; Rozenfeld et al., 2011; Jin et al., 2013), the Maximum-Excluded-Mass-Burning (MEMB) algorithm (Song et al., 2005; Gallos et al., 2008, 2007a,b; Rozenfeld et al., 2011; Jin et al., 2013) and other network clustering and centrality measures to explain the reductive evolution undergone by MLP compared to the closely related MTB genome.

### 2.1. Generating unified MLP and MTB functional networks

Protein–protein functional associations were retrieved from different sources and weighted according to their sources and the technology used to derive them. Functional interactions extracted from the STRING database were used with confidence scores as defined by the STRING schemes, comprised of interactions derived from genomic context (genomic conserved neighbor or gene order, gene fusion events and gene co-occurrence or phylogenetic profiles across genomes), text mining, knowledge from pathway databases, and known experimental interactions. In addition, we derived other interactions from sequence similarity and signatures (shared domains), microarray data (co-expression), Protein Data Bank (PDB; Yellaboina et al., 2009, 2011 and MINT Licata et al., 2012, DIP Salwinski et al., 2004) and Intact (http://www.ebi.ac.uk/intact/) data. We used an information-theory based technique proposed by Mazandu and Mulder (2011a) to derive PPI's from protein sequence similarity and signatures as well as shared domains.

PPI data from MINT, DIP, and Intact were used to predict interologs in MLP based on the premise that orthologs of interacting proteins should themselves interact. Ortholog data were downloaded from Ensembl BioMart at http://www.ensembl.org/biomart/. The Domain–Domain Interactions (DDI) are inferred from Protein Data Bank (PDB) entries and those interactions from PFAM domain definitions predicted by thirteen different methodologies. We extracted DDI's with PFAM ids from the DOMINE website (http://domine.utdallas.edu/cgi-bin/Domine), neglecting self interactions to avoid loops. With the aid of the data containing PFAM ids and their corresponding InterPro ids, we converted those interactions from DDI into their InterPro equivalents, before changing them to UniProt-UniProt protein interaction ids. InterPro data was downloaded from the interPro website (http://www.ebi.ac.uk/interpro) for both MLP and MTB. A uniform score of 0.85 was assigned to all these interactions assumed to be of reasonable quality.

In line with Mazandu et al. (2011), the microarray data for MTB were downloaded from the Standford Microarray Database (SMD), at http:smd.stanford.edu/ and NCBI Gene Expression Omnibus (GEO) at http://www.ncbi.nlm.nih.gov/geo/. However, for MLP, we downloaded only four experiments contained in the GSE17191 series matrix from GEO (http://www.ncbi.nlm.nih.gov/geo/query/acc.cgi?acc=GSE17191). This limited number of microarray experiments prevented us from using the same technique used for MTB, so we used the Pearson correlation coefficient to find co-expressed genes and we inferred interactions between genes for which the correlation coefficient was exactly one.

All functional interactions from these different sources were integrated into a single network. After calculating the confidence score for each functional association protein pair, we computed the combined confidence score C_(*p, q*)_ for interacting proteins *p* and *q* using the formula (Franceschini et al., 2013):

(1) $$C_{\left(p,q\right)}=1-\prod_{s\;=\;1}^{n}\left(1-c_{\left(p,q\right)}^{s}\right),$$

under the assumption of independency, and where *n* is the total number of PPI data sources and $c_{\left(p,q\right)}^{s}$ is the confidence score of a functional association between *p* and *q* predicted using the type of data source *s*. In the two networks, *n* = 11.

### 2.2. Network centrality measures and clustering coefficient

To avoid repetition, we refer the interested reader to Akinola et al. (2013) for a description of some network centrality measures in use, including degree, betweenness, closeness and eigenvector centrality measures. Here, we describe the clustering coefficient of a network useful in the analysis of reductive evolution as it provides an indication of the modular organization of the network. Let *p* be a node with *n*_*p*_ neighbors. The total number of possible edges between *p*'s neighbors is *n*_*p*_(*n*_*p*_ − 1)∕2 (i.e., when every neighbor of *p* is linked with everyone of its other neighbors). Thus, the clustering coefficient of *p* is the ratio of the actual number of edges *a*_*p*_ between *p*'s neighbors to the total number of possible edges. Hence, for undirected networks, the clustering coefficient of a node *p* is defined as Futschik et al. (2007) and Watts and Strogatz (1998):

(2) $$C_{p}=\frac{2a_{p}}{n_{p}(n_{p}-1)}.$$

The clustering coefficient of a node is between 0 and 1. A value zero means there is no clustering and one signifies maximal clustering. For directed networks, the definition is slightly different, i.e., Equation (2) without the factor 2 in the numerator (Watts and Strogatz, 1998). A high clustering coefficient indicates that neighbors of a node are likely to interact with each other (Futschik et al., 2007). The clustering coefficient does not depend on the size of the network (Barabási and Oltvai, 2004) and that is why we are using it in this work to compare networks. The average clustering coefficient on the other hand depends on the number of nodes and edges in the network, describes the overall ability of nodes in the network to form clusters and is defined as Barabási and Oltvai (2004):

(3) $$\bar{C}=\frac{1}{n}\sum_{p\;=\;1}^{n}C_{p}.$$

### 2.3. Fractal, self similarity, and renormalization

In this section, we describe the terms fractal and self similarity as used in the MEMB algorithm for taking different snapshots of a large network and apply this idea to gain an understanding of reductive evolution. Mandelbrot (1986) defined a fractal by making use of the term self-similarity as follows. A set is self-similar if it can be broken into arbitrary small pieces, each of which is a replica of the entire set (Kraft, 1995; see also Engelking, 1978; Mandelbrot, 1982).

**Definition:** *A fractal is a shape made of parts similar to the whole.* There are two methods for computing the fractal dimension of a network: box covering and cluster growing methods. In the cluster growing method, a random node is chosen and a cluster is grown such that the nodes are L_*B*_ distance apart. Moreover, the distribution of the mass in the boxes is exponential with L_*B*_ (Gallos et al., 2007a). The results obtained using this method are biased because of the presence of hubs, since the same hub appears in almost all the boxes. For the purpose of this work, we will base our computations on the box covering method.

Whenever the box covering method is applied to a network and especially (Equation 4), the resulting covering can result in a fractal or non fractal network (Gallos et al., 2007a). In the case of the fractal network, the fractal dimension *d*_*B*_ is finite, they are less compact because hubs are connected with non-hubs and there is a strong hub-hub “repulsion.” Examples of fractal networks are protein–protein interaction networks or metabolic networks, the World Wide Web (WWW) and social networks. Non-fractal networks on the other hand have an infinite fractal dimension, are very compact networks, hubs are connected with hubs and there is a strong hub-hub “attraction.” Examples are the internet at the router level and models based on uncorrelated preferential attachment. Fractality influences the robustness, transport and modularity of a network. Fractal networks are robust against targeted attacks because of the strong hub and non-hub connections and, fractality can be linked with transport in networks. A scaling theory on transport was developed and some important exponents that describe flows in networks were given in Gallos et al. (2007b). In addition, fractality is related to modularity because boxes are synonymous with modules (Gallos et al., 2007a).

Let *G* be a network tiled or covered with box sizes L_*B*_. A box is a set of nodes such that all distances L between any two nodes *p* and *q* inside the box are less than L_*B*_ (Song et al., 2007). Mathematically, a box can be defined as

(4) $$B=\{p,q\in P:L=|p-q|<L_{B}\},$$

where *P* is the set of nodes. Let *N*_*B*_ be the minimum number of boxes needed to cover the whole network *G*. It is trivial to note that if the box size L_*B*_ equals one, then *N*_*B*_ is the total number of nodes in the network (Song et al., 2007). For a given box size L_*B*_, the aim of the box covering algorithm is to find the minimum number of boxes *N*_*B*_(L_*B*_) needed to cover the entire network (Song et al., 2007) such that Equation (4) is satisfied (Song et al., 2005). The fractal dimension *d*_*B*_ describes the self similarity property between different topological scales of the network (Jin et al., 2013). The box size, L_*B*_ and the fractal dimension *d*_*B*_ are related by Jin et al. (2013):

(5) $$N_{B}(L_{B})\sim L_{B}^{-d_{B}}.$$

Every node is covered once.

Once the network is covered, a new network is created known as the renormalized network formed by replacing each box by a node (Song et al., 2005). If there exists at least an edge between any two boxes, then they are connected. The network is renormalized again and again until only one node is left. Scale free networks satisfy the following degree probability distribution, P(*k*), approximating power-law property: that is, for each protein degree *k*,

(6) $$P(k)\sim k^{-\gamma },$$

where γ is the degree exponent. Similarly, renormalized networks have a degree probability distribution P(*k*′) (Song et al., 2005):

(7) $$P(k)\to P(k\prime )\sim {\left(k\prime \right)}^{-\gamma },$$

with *k*′ representing the degree of protein in the renormalized network. Note that unless otherwise stated, prime denotes quantities in the renormalized network; as used in Rozenfeld et al. (2011) and Gallos et al. (2008). Renormalization Group (RG) analysis is a technique that allows one to observe a network at different topological scales. This is because, it transforms the original network into successive smaller copies of itself (Gallos et al., 2012) which can reveal distinct characteristics that are difficult to observe from the original network. In addition, the RG analysis can be used to study how a network evolves and most importantly the evolution of biological networks which is the crux of this paper. An illustrative diagram can be found in Figure 3 of Gallos et al. (2007a).

For a given box size and after a network has been renormalized, the average mass of the boxes used in covering the network 〈*M*_*B*_(L_*B*_)〉 is defined (Song et al., 2005) as:

(8) $$\langle M_{B}(L_{B})\rangle \equiv \frac{N}{N_{B}(L_{B})}=L_{B}^{d_{B}},$$

where *N* is the total number of nodes in the network. Further, the degree *k*′ of the renormalized and the degree *k* of the unrenormalized network satisfy a scaling relationship

(9) $$k\prime =s(L_{B})k,$$

and the scaling factor (*s* < 1) (Song et al., 2005) is related to the box size L_*B*_ by

(10) $$s(L_{B})=L_{B}^{-d_{k}},$$

where *d*_*k*_ is the degree exponent showing how the boxes are connected to each other (Gallos et al., 2007a) or describing how the degree of a node changes during renormalization. From Song et al. (2005), it was stated that for a given L_*B*_, $N^{\prime }=N_{B}\left(L_{B}\right)$, that is, the number of boxes needed to cover the network equals the number of nodes in the renormalized network. This means Equation (8) reduces to:

(11) $$\frac{N}{N\prime }=L_{B}^{d_{B}}.$$

Using the relationship between *n*(*k*) and *n*′(*k*′) as presented in Song et al. (2005):

(12) n(k)dk=n′(k′)dk′,

and the fact that *n*(*k*) = *N*P(*k*) and *n*′(*k*′) = *N*′P(*k*′), then:

(13) NP(k)dk=N′P(k′)dk′.

Upon making the right substitutions for P(*k*), P(*k*′) and using Equation (9),

$$Nk^{-\gamma }dk=N\prime {\left(sk\right)}^{-\gamma }dk\prime .$$

After simplifying and using Equation (9), *Ndk* = *N*′*s*^−γ^*dk*′ and

(14) $$N=N\prime s^{\left(1-\gamma \right)}.$$

Now, *N*′ = *Ns*^(γ−1)^, and by dividing both sides by *N*′ using Equation (11), we have

$$1=s^{\left(\gamma -1\right)}L_{B}^{d_{B}}.$$

But, $s\left(L_{B}\right)=L_{B}^{-d_{k}}$, hence $1=L_{B}^{d_{B}+d_{k}-\gamma d_{k}}$. After applying the laws of indices, it is easy to see that

(15) $$\gamma =1+\frac{d_{B}}{d_{k}}.$$

A box is “compact” if there is no node in the network that can be included in it. In the same vein, a box is “connected” if any node in the box can be reached from any other node in the box and disconnected otherwise (Song et al., 2005).

**Definition 2:** *Given a central node, the box radius r*_*B*_ *is defined as the maximum distance from the central node*.

The box size and box radius are related by

(16) $$L_{B}=2r_{B}+1.$$

This relationship holds for random configurations but fails when the nodes are in a circle (Song et al., 2005).

### 2.4. Maximum-excluded-mass-burning (MEMB) algorithm

As described in Song et al. (2007), there are three methods used to cover a network using the box diameter defined above. The methods are the greedy, random and the Compact-Box-Burning (CBB) algorithms. However, it is still possible to cover a network using the box radius *r*_*B*_ and this is the main idea behind the MEMB algorithm. A box in this case is defined as nodes which are within a radius *r*_*B*_ from a central node. Though the algorithm is not optimal for scale free networks because of the presence of hubs, it gives the same fractal dimension *d*_*B*_ as the greedy and CBB algorithms and it is the easiest to implement. For scale free networks, in Song et al. (2007) (Figure 9), it was shown that burning with the radius from non-hubs as central nodes is worse than burning from hubs. The algorithm makes use of the following definition.

**Definition 3:** *The “excluded mass” of a node is the number of uncovered nodes within a chemical distance less than the box radius r*_*B*_.

The first step in the MEMB algorithm is to compute the excluded mass for all uncovered nodes. This is then followed by covering the network with boxes of maximum excluded mass.

1. Mark all nodes as uncovered and non-centers.
2. For every non-central node (including nodes that are covered), compute the excluded mass and choose the node *s* having the maximum excluded mass as the next center.
3. Mark all the nodes with chemical distance less than *r*_*B*_ from *s* as covered.
4. Repeat the last two steps until all nodes are either centers or covered.
5. The number of selected centers correspond to *N*_*B*_.

Throughout this paper, all networks were drawn using PINV (Salazar et al., 2014) (http://biosual.cbio.uct.ac.za/pinv.html). In the results, an 'insertion' means the addition of a non-orthologous protein to a protein's neighborhood.

### 2.5. Analysis of MLP pseudogenes in MTB network

We extracted 1115 pseudogenes from MLP with their start and end positions from NCBI (Benson et al., 2009; Sayers et al., 2009). Fasta sequences for these pseudogenes were downloaded from the NCBI website using the Biopython package (Chapman and Chang, 2000; Cock et al., 2009). We ran the MLP pseudogene nucleotide sequences against the protein sequences of MTB using BLASTX (Altschul et al., 1990; Gish and States, 1993; Madden et al., 1996) with an *E*-value cutoff of 10^−10^.

## 3. Results and discussion

**Figure 1:**
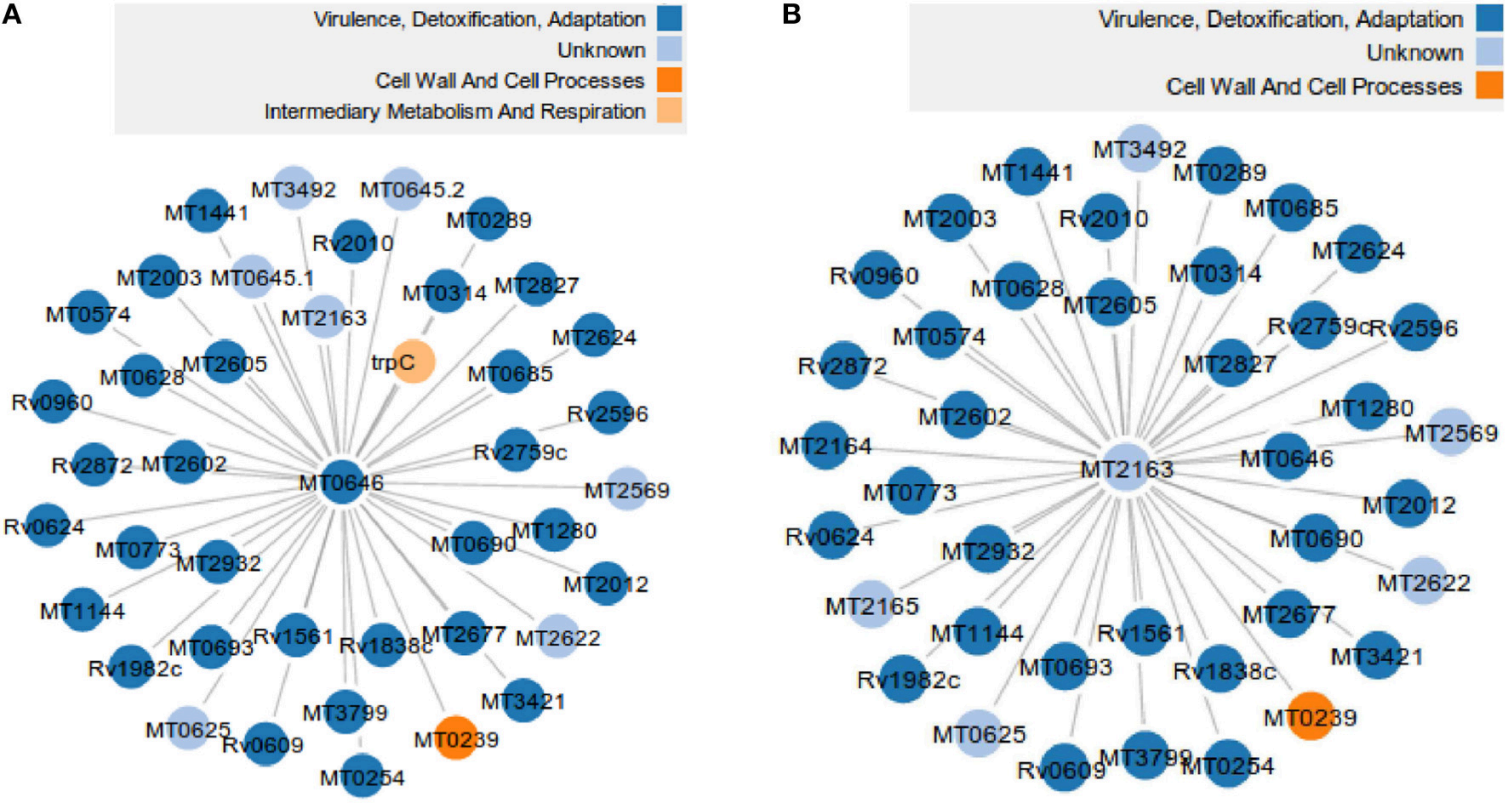
**Two out of 59 hub proteins and their 80 neighbors in the MTB network that have been deleted/have no orthologs in MLP**. **(A)** The probable ribonuclease hub protein “O07760” (MT0646) in MTB and its 40 neighbors absent from MLP. **(B)** The putative uncharacterized hub protein “Q7D7I7” (MT2163) in MTB and its 40 neighbors absent from MLP.

Comparison of orthologs between MTB and MLP shows that there are 2859 proteins unique to the MTB network i.e., not present in the MLP network, 135 unique to MLP and they share 1277 proteins in common (Mulder et al., 2014). From the functional MTB PPI used in Akinola et al. (2013) containing 59,919 edges (functional interactions), 4136 nodes (proteins) and 201 hubs, there are 25,916 functional interactions which are unique to MTB. This corresponds to 43.2% of the total number of functional interactions in the MTB network. This suggests that MLP has lost 2859 proteins in its genome and 25,916 interactions from its PPI network even though it shares a common ancestor with MTB (Table 1). Out of the 201 hub proteins in the MTB network, 164 have no orthologs in MLP. A close look at each of these 164 hubs reveals that 59 also have no orthologous neighbors; that is, all their neighbors in MTB have been deleted in MLP. If we remove these 59 hub-proteins and their edges from the MTB network, we have 3972 (94.6%) proteins out of 4136 and 55,860 (93.2%) functional interactions. However, the removal disconnected the entire MTB network into 175 connected components and the percentage of the largest component became 93.6%. We give two examples of proteins in MTB each with 40 neighbors which have been lost/deleted in MLP: “Q7D7I7” (MT2163) with UniProt description “Putative uncharacterized protein” and “O07760” (MT0646) “Probable ribonuclease.” Figure 1 shows that most of the neighbors belong to the ‘virulence, detoxification and adaptation’ functional class and these proteins are absent from MLP.

**Table 1:** **Comparing network parameters and values in MTB and MLP subnetworks**.

**Parameters**	**Values**	
	**MTB**	**MLP**
Number of proteins (Nodes)	2859	135
Number of functional interactions (Edges)	25916	143
Number of hubs	281	16
Density	0.0065	0.0488
Average degree	20	4
Average shortest path length	4.2077	1.8974
Average clustering coefficient	0.5610	0.4997
Number of connected components	42	14
% of Nodes in largest component	94.5%	12.5%

The subnetworks comprise proteins unique to the MTB or MLP networks.

After filtering the 59 proteins by clustering coefficients with degrees greater than five as the cut-off, we found 16 proteins. We also noted that the betweenness centralities of these 59 proteins are very high. Only three protein's betweenness are below 4000 as shown in Figure S1. This shows that these proteins play crucial roles in the survival and flow of information in the MTB network. As shown in Figure S2, we see that only three of these proteins have GC contents less than the average GC content for the MLP genome, quite a number are above this threshold (57.7% and above the lower blue line). This shows that these proteins are GC rich and their absence from the MLP proteome might have contributed to the reduced genome and GC content of MLP. However, the MTB proteome is generally GC rich and these 59 proteins are just examples, because any random set of proteins from the MTB proteome shows a similar trend.

As mentioned in the materials and methods section, the clustering coefficient does not depend on the size of the network, therefore, we computed the clustering coefficient of each of the 1277 orthologous proteins in both organisms to see if there is a correlation between them. We calculated the Spearman's correlation coefficient and *p*-value between their clustering coefficients as 0.3093 and 1.0 × 10^−29^, respectively. The mean±standard deviation are 0.4853 ± 0.3194 for MLP and 0.4402 ± 0.2471 for MTB, *r*^2^ = 0.0849. The correlation coefficient shows that though the 1277 proteins have orthologs in both MLP and MTB, there is a low linear correlation (0.3093) between their clustering coefficients. To gain further insight into the low correlation, we looked at the clustering coefficients of orthologs that are either hubs in MTB or hubs in MLP. We counted 127 such candidate proteins and compared their clustering coefficients by plotting them for each protein id. Results (not shown) indicate that the clustering coefficients were randomly distributed.

Out of the 1277 orthologs common to both organisms, 1188 proteins (76.1%), have the same functional classes while 89 differ. The distribution of the number of proteins corresponding to each conserved functional class is shown in Table 2. Two subnetworks comprising the 392 proteins belonging to the intermediary metabolism and respiration functional class, have 2911 edges out of the 59,919 edges in the MTB network and 2946 edges out of the 20,742 edges in the MLP network. Interestingly, the average clustering coefficient of the two subnetworks are almost the same, 0.4141 for MTB and 0.4191 for MLP. However, the two subnetworks were “highly” disconnected (considering the number of edges) with the number of connected components being 14 and 17 for MTB and MLP, respectively.

**Table 2:** **The distribution of the number of proteins with conserved functional classes in the two mycobacteria**.

**Functional classes**	**Number of proteins**
Intermediary metabolism and respiration	392
Cell wall and cell processes	242
Unknown/conserved hypotheticals	216
Information pathways	168
Lipid metabolism	79
Regulatory proteins	42
Virulence, detoxification, adaptation	42
pe/ppe	5
Insertion seqs and phages	2
Pseudogene	0
Total	1188

We examined the 89 proteins with diverged functional classes among the orthologs in the two species and the results are presented in Table S1. The table shows that 10 proteins in MLP have changed functional class from “conserved hypotheticals” to “intermediate metabolism and respiration” in MTB. In the same vein, 2 proteins in MLP belonging to “conserved hypotheticals” functional class have changed to “cell wall and cell processes” in MTB. These differences may reflect misannotations or the less well annotated status of the MLP proteome compared to MTB.

**Figure 2:**
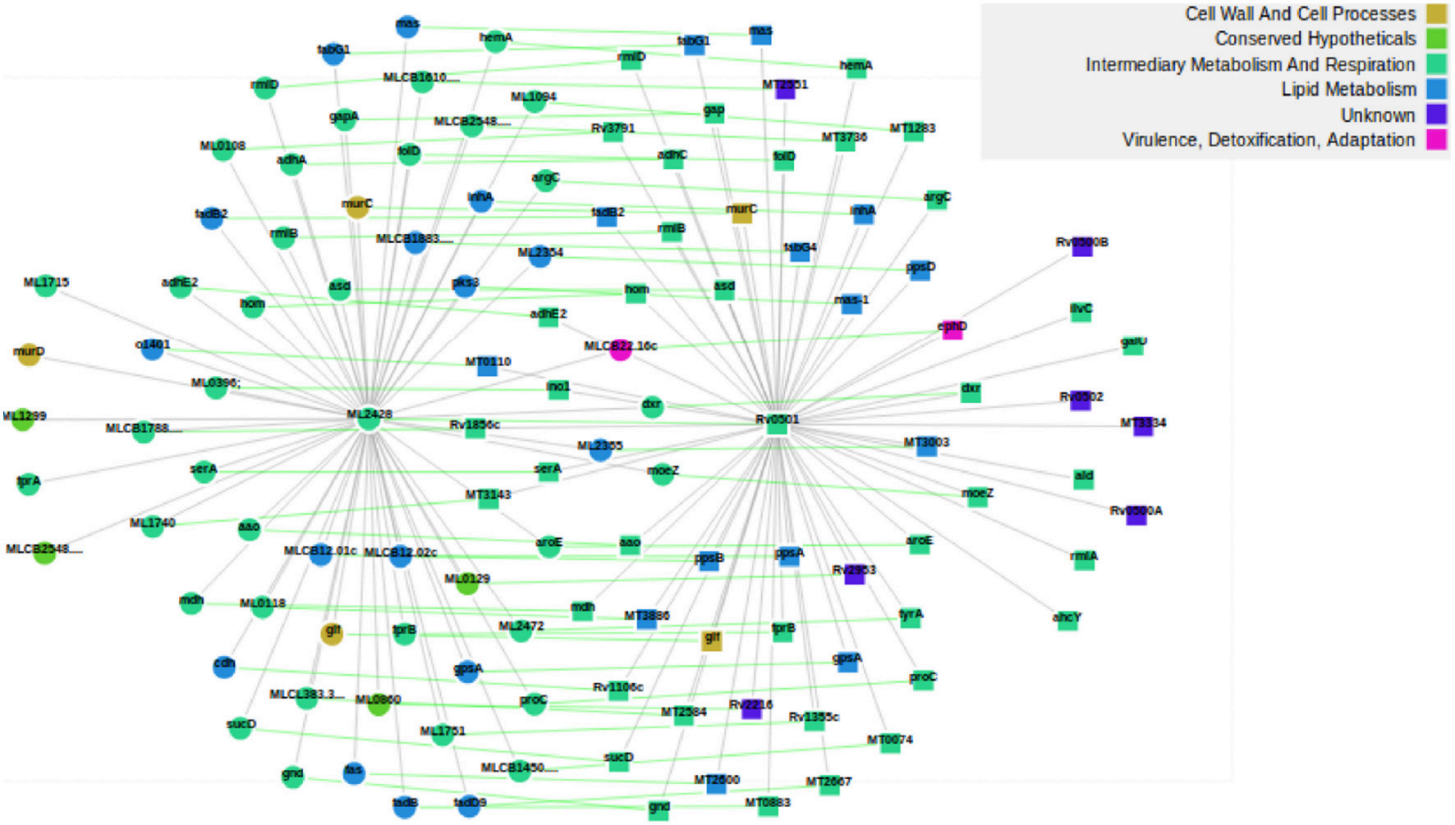
**The two proteins Q9CB57/ML2428 in MLP (left) and P0A5D1/Rv0501 (right) in MTB, and their respective 58 and 62 neighbors**. As shown by the green lines, 53 proteins are direct ortholog neighbors in both. In this example, we used the ortholog network.

**Figure 3:**
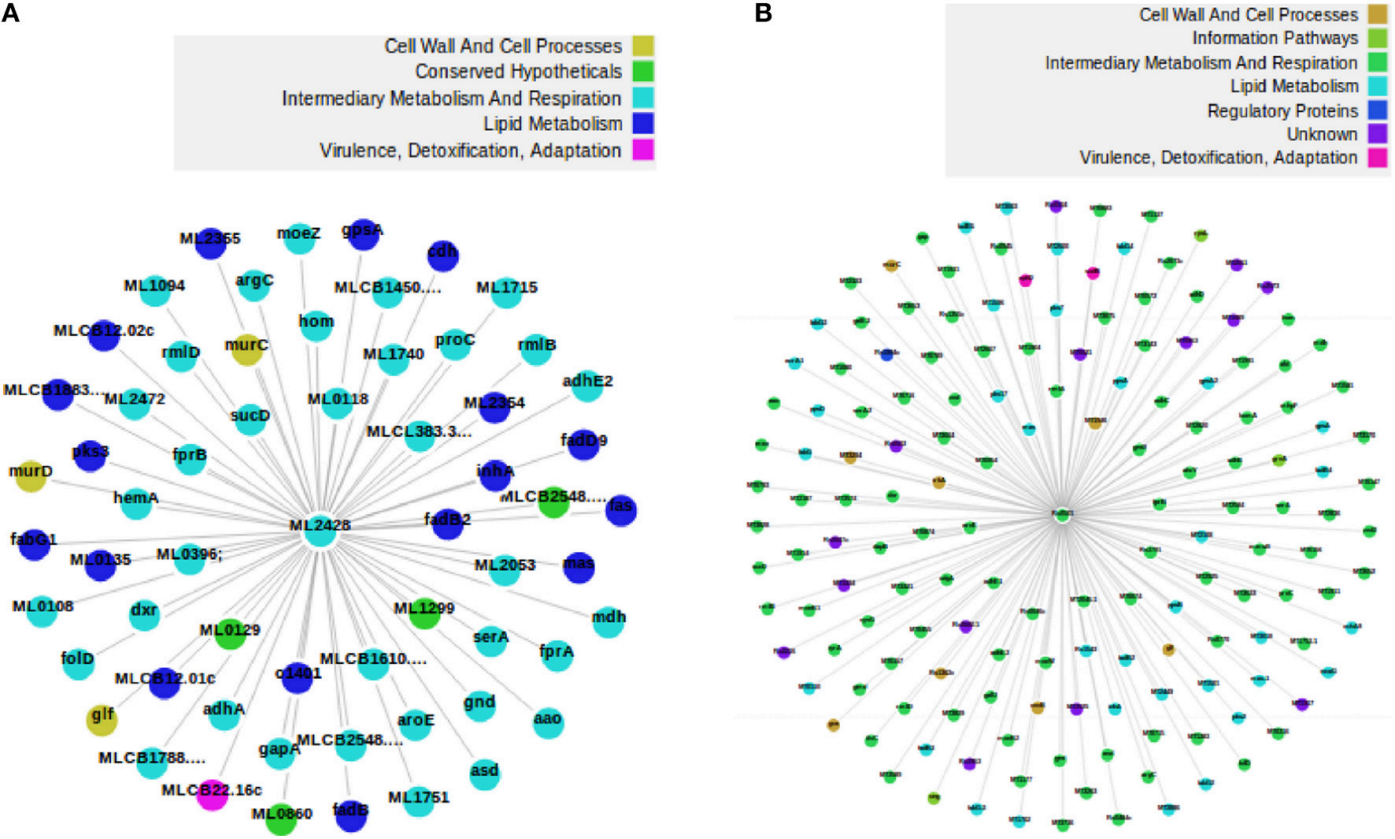
**An example of a deletion in MLP**. The two proteins are orthologs in MLP (left) and MTB (right), respectively, but Q9CB57 has 90 of its neighbors deleted with respect to its ortholog network. Fifty three proteins are direct ortholog neighbors in both. **(A)** The protein Q9CB57 in MLP and its 60 neighbors comprising 2 non-orthologs and 58 orthologs. **(B)** The protein P0A5D1 in MTB and its 152 neighbors. Among the neighbors, 90 are non-orthologs while 62 are orthologs. We drew the two figures from the full network.

We subdivided the 1277 orthologs into three groups based on the number of neighbors; such that the number of neighbors in MLP are either less than, equal to or greater than the number of neighbors in MTB. 882, 18, and 377 candidate proteins in MLP have neighbors less than, equal to, and greater than their corresponding number of neighbors in MTB, respectively. This categorization is important because for each orthologous pair, we identified where insertions or deletions (indels) took place in each of the networks. Specifically, we are interested in cases where proteins have been deleted in the MLP proteome as a case for reductive evolution. For example, let (*p, q*) be an orthologous pair of proteins; where *p* is from the MLP network and *q* from the MTB network. If the number of neighbors of *p* are less than the corresponding number of neighbors of *q*, then, this represents (the “less than” case) a case in which proteins have been deleted from the MLP network. This is one of the functional network analysis approaches for detecting genome reduction. Table S3 shows properties of the subnetworks formed from just the 882 orthologous candidate proteins where MLP proteins have fewer neighbors than their MTB counterparts. Two examples to illustrate deletions in MLP are given in Figures S3, S4 and Figures 2, 3. As shown in Figure S3, the two orthologous proteins: O32890 (MLCB1779.30) “the putative acyl-CoA dehydrogenase protein” in MLP and P95187 (fadE24) the “probable acyl-CoA dehydrogenase protein” in MTB have six each in the ortholog subnetwork of which three are direct orthologs. O32890 had two protein neighbors inserted which have no ortholog in MTB, while 41 such neighbors were inserted for P95187. This accounts for the 8 neighbors of the MLP protein and 48 of the MTB protein as illustrated in Figure S4. In the second example, Figure 2 shows the ortholog network of the two proteins: the “possible glucose epimerase/dehydratase protein” Q9CB57 (ML2428) in MLP and the “uncharacterized protein” P0A5D1 (Rv0501) in MTB with their 58 and 62 neighbors, respectively, while Figure 3 shows that 90 proteins have been deleted from the MLP protein's neighbors.

**Figure 4:**
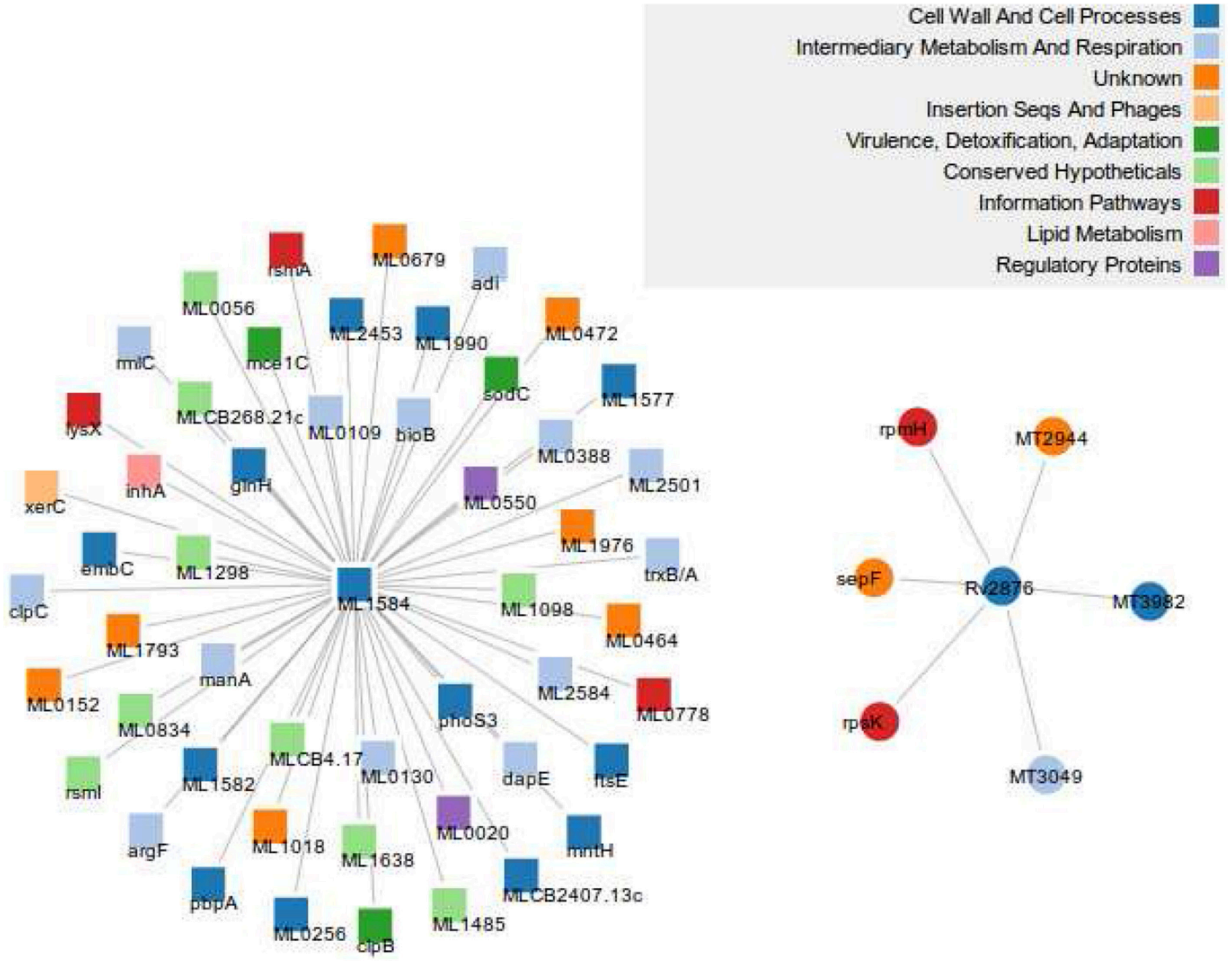
**An example of an insertion/deletion in MTB**. Q9CBU2 in MLP (left) and its 51 neighbors, and its ortholog Q10802 (right) in MTB together with 6 non-orthologous neighbors. Forty out of the 51 neighbors have orthologs in MTB, but are part of other subnetworks. This shows that 11 proteins have been inserted in the MLP network while the MTB network has 6 proteins inserted. There are no direct ortholog neighbors in both subnetworks. We used the full network in creating this figure.

In the same vein, the “greater than” case means MTB has experienced some loss of proteins on the one hand or MLP has undergone some insertions on the other. A closer look at the subnetworks of both networks consisting of these 377 orthologous proteins shows that there are 1267 and 8139 edges in MTB and MLP, respectively, constituting 2.1 and 39.2% of the total number of edges in their respective networks. We give two examples in Figure S5 and Figure 4. In the first example, Figure S5 shows Q49999 (ML1037) the putative uncharacterized protein in MLP and its 30 neighbors in the ortholog subnetwork and O07185 (MT2757), the “CBS domain protein” in MTB with its 10 inserted non-ortholog and 5 orthologous neighbors. The two proteins belong to the intermediary, metabolism and respiration functional class. Similarly, Figure 4 shows the neighbors of the two orthologous proteins Q9CBU2 (ML1584) in MLP and Q10802 (Rv2876) in MTB. Both have UniProt description “uncharacterized protein” and belong to the same functional class, “cell wall and cell processes.” Another example is shown in Figure S5 in Supplementary Material. While MLP has more neighbors than MTB, some of the MTB neighbors are proteins that are not present in MLP, i.e., they have either been lost by MLP or gained by MTB.

Furthermore, we checked for proteins in both networks that have the same degree and found a total of 18 candidate proteins. As shown in Table S2, 12 of them have the same functional classes. A Pearson correlation test reveals that there is a statistically significant correlation between their clustering coefficients with correlation coefficient = 0.51, *r*^2^ = 0.2631, *p*-value = 0.029. One point of interest is that though the proteins ‘Q9CDE8’ and “P71580” in MLP and MTB, respectively, belong to the same functional class and each have one neighbor, their neighbors are not orthologs. A similar result holds for “Q49803” in MLP and “P65300” in MTB. Therefore, they are connecting to different proteins.

Since the sizes of the two networks are different, we considered the set of 1277 orthologs in both mycobacterial species to identify ancestral proteins and determined their significance in their respective networks. To do this, we used the MEMB algorithm discussed in the materials and methods section by applying it to the largest connected components of the two subnetworks, with the distance L between two proteins defined as the length of the shortest path between these proteins in the functional network. The results of the network topologies are presented in Table 3. We found 15 proteins in the MLP PPI subnetwork and 9 in the MTB PPI subnetwork having different box radii ranging from one to three. We did this to see how the degree varies with the box radius and noted that for most of the proteins with box radii less than or equal to 3 in both subnetworks, the degree decreases as the box radii increases. The results are presented in Figures S6, S7. The protein “P45486” in the MLP subnetwork (Figure S6) and “O06620” in the MTB subnetwork (Figure S7) are orthologs and belong to the functional class, “intermediary metabolism and respiration.” Conservation of functional classes is expected since protein hubs experience stronger selective constraints than non-hub proteins (Kaçar and Gaucher, 2013) in the functional network as they are essential for the survival of the organism (Mazandu and Mulder, 2011b). Thus, biological functions of these hubs proteins tend to be evolutionarily more conserved than the others. The MEMB algorithm for *r*_*B*_ = 1 applied on the largest component of both subnetworks re-confirms the fact that both organisms descended from a common ancestor as shown in Table 4, this is because both organisms have almost the same number of ancestral orthologous proteins, 193 and 182 for MLP and MTB, respectively. Similarly, both organisms have almost the same number of ancestral functional, interactions viz 2670 for MLP and 2787 for MTB.

**Table 3:** **Subnetwork topologies computed using the largest connected components**.

**Parameter**	**MLP**	**MTB**
*d*_*B*_	3.4	3.5
*d*_*k*_	2.5	3.7
$\frac{d_{B}}{d_{k}}$	1.4	0.9
$\gamma =1+\frac{d_{B}}{d_{k}}$	2.4	1.9
No. of nodes in subnetworks	1277	1277
No. of edges in subnetworks	18223	13047
No. of nodes in Largest component	1239	1254
No. of edges in Largest component	18207	13047
% of edges in Largest component	99.8	100
% of nodes in Largest component	97.0	98.1

We removed non-orthologous MTB and MLP proteins from the two networks to ensure each subnetwork has 1277 proteins.

**Table 4:** **Number of proteins at each stage of the renormalization process for *r*_*B*_ = 1 in the two mycobacteria**.

**Parameter**	**Stage 1**		**Stage 2**		**Stage 3**	
	**MLP**	**MTB**	**MLP**	**MTB**	**MLP**	**MTB**
Number of nodes	193	182	34	20	4	–
Number of edges	2690	2787	351	159	4	–

After blasting the 1115 MLP pseudogene nucleotide sequences against the MTB protein sequences using BLASTX with an *E*−value cutoff of 10^−10^, we obtained 899 proteins in the MTB proteome, 875 of which are in the MTB network and 25 have orthologs in MLP. Only one of the 875 proteins is a hub protein in MTB, this is P95315, which hit the pseudogene ML1054. This provides an indication that some of the MTB proteins which are “pseudogenized” in MLP are well connected proteins in the MTB functional network, thus playing important roles. To confirm this, we compute the average network centrality scores and check whether the values for MTB proteins with pseudogenes in MLP are higher than those of the remaining proteins in the MTB network. The average eigenvector, betweenness, closeness and degree centralities of the 875 MTB proteins with pseudogenes in MLP are 0.0047, 5377.38, 0.2837, and 32, respectively. These numbers surpass those of the remaining 3261 MTB proteins which are 0.0031, 5274.64, 0.2720, and 28, which shows that these proteins on average play crucial roles in the MTB network. To ensure that these average values are more than expected by chance, we randomly chose 10 independent sets of 875 proteins in the 3261 MTB proteins. After computing the average network centrality values for each set, the means viz average eigenvector, betweenness, closeness and degree centrality values are 0.0034, 5244.23, 0.2747, and 29, respectively. This suggests that some of the 875 MTB proteins with pseudogenes in MLP are greater than the average network centrality values, indicating that these proteins are important in the MTB system, helping in maintaining the “small world” property and in quickly exhibiting a qualitative change in response to the system perturbations.

Finally, we looked at the functional class in which the 2859 proteins unique to MTB are involved. The distribution of these proteins per functional class is shown in Table 5. Table 5 indicates that more than 90% of proteins involved in “*insertion seqs and phages”* and “*PE/PPE”* functional classes are specific to MTB. These proteins are probably key players in mediating genome rearrangements and deletions (Fang et al., 1999), and may play an important role in immunogenicity (Mazandu and Mulder, 2011b). Some of these proteins are pseudogenes in MLP, which are non functional genes that are still functionally active in MTB as observed above. These genetic differences provide a sign of selective pressure, which altered genes in MLP, possibly for adaptation to its environments during infection and transmission. This has potentially influenced pathogenesis and immunity, and has defined the genotype and intracellular lifestyle differences between these two pathogens, which remarkably reflect on each organism's pathogenicity and disease phenotype.

**Table 5:** **The distribution per functional class of 2859 proteins unique to MTB (PUM) and that of 875 with pseudogenes in MLP (PPM)**.

**Functional class**	**Number of PUM**	**Number of PPM**
Cell wall and cell processes	368	139
Intermediary metabolism and respiration	476	229
Information pathways	72	36
Insertion seqs and phages	77	12
Lipid metabolism	148	83
Virulence, detoxification, adaptation	133	20
Regulatory proteins	132	57
Unknown	1311	291
pe/ppe	142	8
Total	2859	875

## 4. Conclusions

In this study, we analyzed reductive evolution based on functional interaction networks, focusing on the MLP genome to reveal different biological features that are able to explain a massive reductive evolution undergone by MLP in comparison to the closely related MTB genome. Out of 201 hubs found in the MTB functional network, we identified 164 without orthologs in MLP, of which 59 have no orthologous neighbors either. That is, 59 proteins and their 257 neighbors (formed by the union of all neighbors, i.e., without multiplicities) were deleted from the MLP proteome during reduction in its genome. The GC content of most of these 59 proteins were above the GC content of MLP itself. Furthermore, out of the 1277 orthologous proteins in both networks, we identified 1188 and 89 proteins with conserved and divergent functional classes, respectively, in both organisms. This may be due to the state of annotation in each. It is also important to note that due to the divergence of MLP, the orthologs may not have exactly the same functions. For example, Patil et al. (2011), compared the activities of MLP RecA protein with those of MTB RecA protein after cloning, purifying and over-expressing it. They found that while at the amino acid level the RecA protein were 91% identical, they are functionally different in both micro-organisms.

In order to identify instances where MLP suffered insertions/deletions (indels), we further classified the 1277 proteins based on their degrees into those in which MLP had a lower number of neighbors compared to MTB, equal number of neighbors and those in which MLP had a higher number of neighbors compared to MTB; we found 882, 18, and 377 proteins, respectively. For each orthologous pair among the 377 proteins, we found instances where the MTB network had insertions of novel proteins (not present in MLP), or where its MLP counterpart had suffered massive deletions.

Besides the deletions providing a means of reductive evolution in MLP, the reduction can also by viewed as a corresponding lack of insertions of orthologs compared to MTB. Starting with the 1277 orthologs in both organisms, while 2859 proteins were added to MTB, only 135 were added to MLP. The inserted proteins contributed 25,916 and 143 edges to the MTB and MLP networks, respectively. This work provides a quantitative model for mapping reductive evolution and protein–protein functional interaction network organization in terms of roles played by different proteins in maintaining the stability and the structure of the system. The MEMB algorithm for *r*_*B*_ = 1 applied on the largest component of both ortholog subnetworks re-confirms the fact that both organisms descended from a common ancestor because both organisms have almost the same number of ancestral proteins: 193 and 182 for MLP and MTB, respectively. In the same vein, both organisms have almost the same number of ancestral functional interactions viz 2690 for MLP and 2787 for MTB. Finally, by taking a look at the pseudogenes, we found that the MTB orthologs of the MLP pseudogenes tended to have higher than average centrality measures. The removal of these potentially important proteins in MLP may be the cause of the limited host range and growth potential outside of these hosts in MLP.

## Author contributions

Conceived the experiments: NM. Analyzed the model and performed the experiments: RA, GM. Analyzed the data: RA, GM, NM. Contributed reagents/materials/analysis tools: RA, GM, NM. Wrote the paper: RA, GM, NM. Finalized the manuscript: NM. Read and approved the final manuscript: RA, GM, NM.

## Funding

The authors appreciate financial support received from the National Research Foundation (NRF) South Africa (grant number 86934). Some of the authors are funded in part by Government of Canada via the International Development Research Centre (IDRC) through the African Institute for Mathematical Sciences—Next Einstein Initiative (AIMS-NEI).

### Conflict of interest statement

The authors declare that the research was conducted in the absence of any commercial or financial relationships that could be construed as a potential conflict of interest.
