# Supplementary material for: A Quantitative Approach to Analyzing Genome Reductive Evolution Using Protein–Protein Interaction Networks: A Case Study of Mycobacterium leprae
Source: Front Genet. 2016 Mar 29;7:39. doi: 10.3389/fgene.2016.00039 (PMC4809885; doi:10.3389/fgene.2016.00039)
Supplement: Supplementary file 1 [file Akinola_Supplementary.pdf]

Section S1: Deriving the scoring function used and illustrating its effectiveness

The confidence score of a functional interaction between proteins p and q measures our confidence level in this specific functional interaction and represents the probability or likelihood of the occurrence of this interaction. Assume that n different sources were used to predict this interaction and let $\overline{\mathcal{A}_{p,q}}$ be an event indicating that the functional interaction between proteins p and q could not be inferred from any of these n sources under consideration, that is:

$$\overline{\mathcal{A}_{p,q}} = \bigcap_{s=1}^{n} \overline{\mathcal{A}^{s}_{p,q}} \tag{1}$$

with $\overline{\mathcal{A}^{s}_{p,q}}$ the event indicating that the functional interaction could not be retrieved using the source s. Under the assumption that sources are independent, the probability $\mathbb{P}\left(\overline{\mathcal{A}_{p,q}}\right)$ of the event $\overline{\mathcal{A}_{p,q}}$ is given by:

$$\begin{aligned} \mathbb{P}\left(\overline{\mathcal{A}_{p,q}}\right) &= \mathbb{P}\left(\bigcap_{s=1}^{n} \overline{\mathcal{A}^{s}_{p,q}}\right) \\ &= \prod_{s=1}^{n} \mathbb{P}\left(\overline{\mathcal{A}^{s}_{p,q}}\right) \\ &= \prod_{s=1}^{n} \left(1 - \mathbb{P}\left(\mathcal{A}^{s}_{p,q}\right)\right) \end{aligned} \tag{2}$$

where $\mathcal{A}^{s}_{p,q}$ is the event indicating that the functional interaction is retrieved using the source s and thus $\mathbb{P}\left(\mathcal{A}^{s}_{p,q}\right) = c^{s}_{(p,q)}$ with $c^{s}_{(p,q)}$ the confidence score of a functional association between p and q predicted using the source s. Thus, the combined confidence score $\mathcal{C}_{(p,q)}$ for interacting proteins p and q, which is the probability of the event $\mathcal{A}_{p,q}$, which indicates that the functional interaction between proteins p and q can be inferred from at least one of the sources, contrary to $\overline{\mathcal{A}_{p,q}}$, is given by:

$$\begin{aligned} \mathcal{C}_{(p,q)} &= \mathbb{P}\left(\mathcal{A}_{p,q}\right) \\ &= 1 - \mathbb{P}\left(\overline{\mathcal{A}_{p,q}}\right) \\ &= 1 - \prod_{s=1}^{n} \left(1 - \mathbb{P}\left(\mathcal{A}^{s}_{p,q}\right)\right) \end{aligned} \tag{3}$$

It follows that:

$$\mathcal{C}_{(p,q)} = 1 - \prod_{s=1}^{n} \left(1 - c^{s}_{(p,q)}\right) \tag{4}$$

Finally, let us illustrate how other scoring functions, such as minimum (min), maximum (max) and average (mean) of different confidence scores may produce biased combined or unified score, and why the scoring function in equation (4) is more realistic. Assume that out of $n = 11$ different data sources, the functional interaction between proteins p and q was predicted from 2 sources out of 11 with confidence scores of 0.200 and 0.130. So, for any other source, the confidence score is assumed to be 0, and it follows that:

– Using the min function, we get $\mathcal{C}_{(p,q)} = \min\{0,0,0,0,0,0,0,0,0,0.200,0.130\}$, which implies that $\mathcal{C}_{(p,q)} = 0.00$, indicating that the confidence score is 0 and this interaction will be ignored in different analyses whereas it was predicted by two different sources.

– Using max and mean, the combined confidence score, $\mathcal{C}_{(p,q)}$, is equal to 0.200 and 0.030. The max function does not reflect the fact that the functional interaction was predicted from two different sources

and the mean function reduces our confidence level.
Intuitively, as this interaction was predicted by two different sources, one expects its confidence level to increase, but instead it is decreasing. This suggests that these scoring functions are not in agreement with what can be expected and show biases by underestimating combined interaction scores in the final network. On the other hand, using the scoring function in equation (4) as used in the paper, we have $\mathcal{C}_{(p,q)} = 0.304$, showing more realistic combined confidence score compared to other scoring functions, and is in agreement with what one expects.

Table S1. Divergence of 89 functional classes in the two mycobacteria.

Mycobacterium leprae strain TN<br>Functional class	*Mycobacterium tuberculosis* strain CDC1551<br>Functional class	Number of Proteins
conserved hypotheticals	intermediary metabolism and respiration	10
conserved hypotheticals	cell wall and cell processes	2
conserved hypotheticals	regulatory proteins	1
conserved hypotheticals	lipid metabolism	1
cell wall and cell processes	unknown	46
cell wall and cell processes	information pathways	1
cell wall and cell processes	intermediary metabolism and respiration	1
intermediary metabolism and respiration	cell wall and cell processes	1
intermediary metabolism and respiration	information pathways	3
intermediary metabolism and respiration	virulence, detoxification, adaptation	1
intermediary metabolism and respiration	lipid metabolism	2
intermediary metabolism and respiration	unknown	7
information pathways	unknown	2
information pathways	intermediary metabolism and respiration	1
lipid metabolism	intermediary metabolism and respiration	3
virulence, detoxification, adaptation	unknown	1
pseudogene	information pathways	1
pseudogene	regulatory proteins	1
regulatory proteins	intermediary metabolism and respiration	1
regulatory proteins	unknown	3
**Total**		89

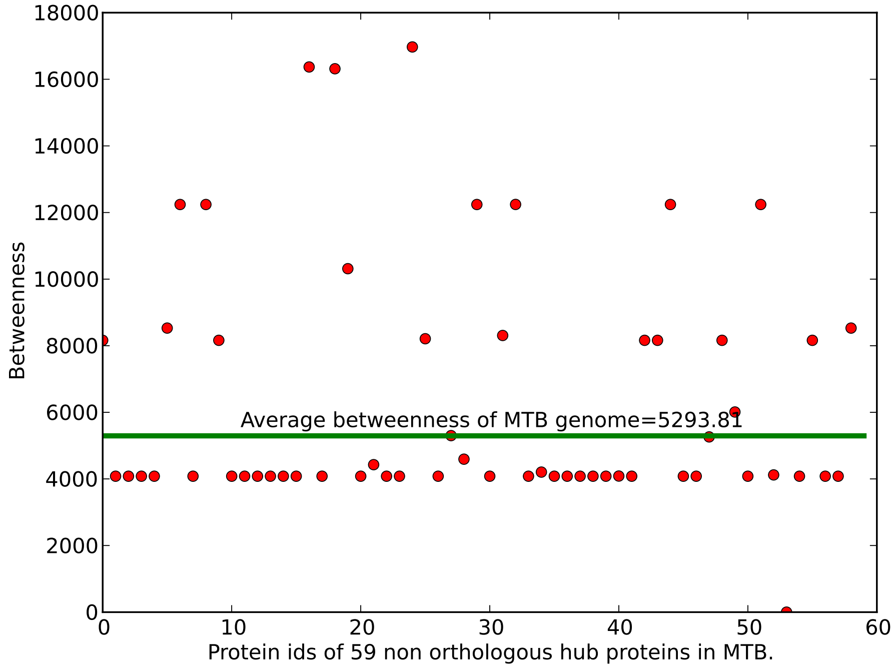

Figure S1: Graph showing high betweenness centralities of the 59 hub proteins in MTB. The green line depicts the average betweenness (5293.81) of the entire MTB genome. A total of 23 proteins are above this threshold.

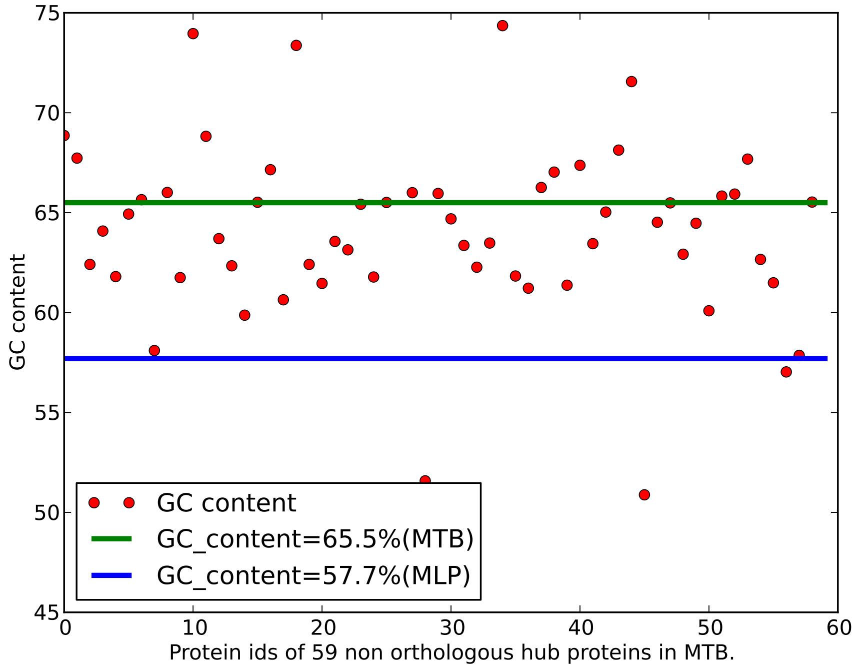

Figure S2: This graph plots the GC content of the 59 hub-proteins in MTB. The upper and lower line represents the guanine plus cytosine (GC) contents for the complete MTB and MLP genomes respectively.

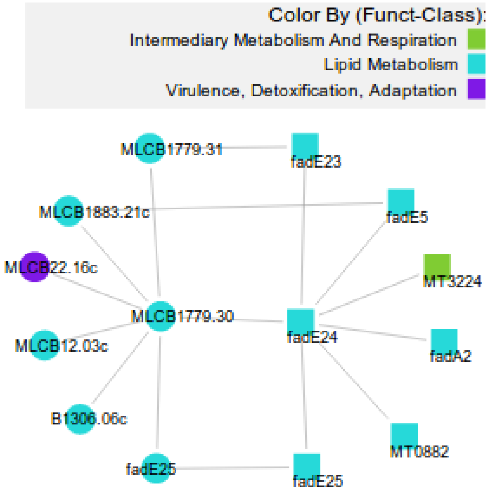

Figure S3: The two orthologous proteins O32890 in MLP (left) and P95187 in MTB (right) with their neighbours in the ortholog subnetwork. Both proteins have the same number of neighbours but only three proteins in both are direct ortholog neighbours (they are linked together by a horizontal line). This figure was extracted from the ortholog network.

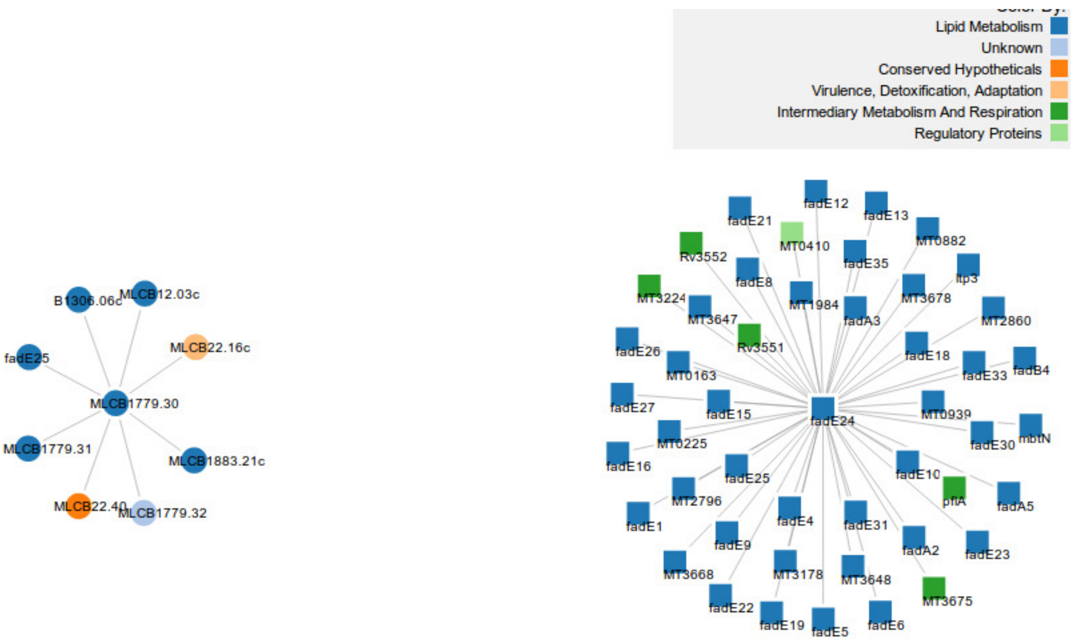

Figure S4: An example of a deletion in MLP. The same orthologous proteins O32890 with its 8 neighbours in MLP (left) and P95187 with its 47 neighbours in MTB (right) from Figure **??** but now in the full network. Two of the MLP protein's neighbours do not have orthologs in MTB while the remaining 6 do have orthologs. 41 out of the 47 neighbours of P95187 have no orthologs in MLP. This shows that approximately 41 proteins have been deleted from the neighbours of O32890 in MLP. There are three direct ortholog neighbours in both as shown in Figure S3.

Table S2. Functional classes of the 18 proteins which have the same degree and are orthologs.

Mycobacterium leprae strain TN<br>Protein	 Functional class	*Mycobacterium tuberculosis* strain CDC1551<br>Protein	 Functional class	Degree	#Ortholog neighbours
Q9Z5I3	cell wall and cell processes	O53933	cell wall and cell processes	5	2
Q9CBV4	cell wall and cell processes	O53946	cell wall and cell processes	4	2
P54134	cell wall and cell processes	Q11013	cell wall and cell processes	2	1
Q49803	cell wall and cell processes	P65300	cell wall and cell processes	1	0
Q49630	cell wall and cell processes	O07802	cell wall and cell processes	2	0
Q9CC55	cell wall and cell processes	O06128	cell wall and cell processes	6	0
Q9CDE8	cell wall and cell processes	P71580	cell wall and cell processes	1	0
Q9CB98	intermediary metabolism and respiration	O69636	intermediary metabolism and respiration	8	5
Q9X7F1	intermediary metabolism and respiration	P66897	intermediary metabolism and respiration	40	15
Q50000	intermediary metabolism and respiration	P0A554	intermediary metabolism and respiration	33	13
O05564	intermediary metabolism and respiration	P65340	intermediary metabolism and respiration	20	11
Q9CCZ3	intermediary metabolism and respiration	P0A5L0	intermediary metabolism and respiration	10	0
Q9CC40	conserved hypotheticals	O06242	unknown	5	0
Q9CCG7	conserved hypotheticals	O05861	unknown	2	1
Q9CC33	conserved hypotheticals	O33186	unknown	2	0
Q9CCG9	conserved hypotheticals	Q6ARF7	unknown	3	1
O69598	conserved hypotheticals	Q7D9Y5	unknown	3	0
Q49834	cell wall and cell processes	Q8VJE1	unknown	2	0
				149	51

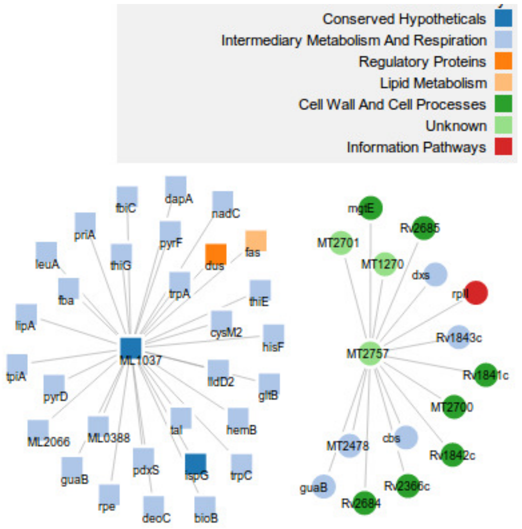

Figure S5: An example of an insertion/deletion in MTB. The two proteins Q49999 (ML1037) and O07185(MT2757) are orthologs of each other. Q49999 (left) in MLP and its 30 neighbours, and O07185 (right) in MTB and its 15 neighbours. All the 30 neighbours of Q49999 have orthologs in MTB while only five are direct ortholog neighbours of O07185 and the rest are not. We used the full network for this example.

Table S3. Comparing network parameters and values in subnetworks of MTB and MLP. The subnetworks comprise proteins that are orthologs in both MTB and MLP, but with number of neighbours of each MLP protein less than the corresponding number of neighbours of each MTB protein. 882 proteins belong to this category.

Parameters	Values	
	MTB	MLP
Number of proteins (Nodes)	882	882
Number of functional interactions (Edges)	7791	5439
Number of hubs	34	104
Density	0.0208	0.0155
Average degree	18	12
Average shortest path length	3.2034	3.7669
Number of connected components	4	24
Average clustering coefficient	0.4463	0.4195
% of Nodes in largest component	96%	88%

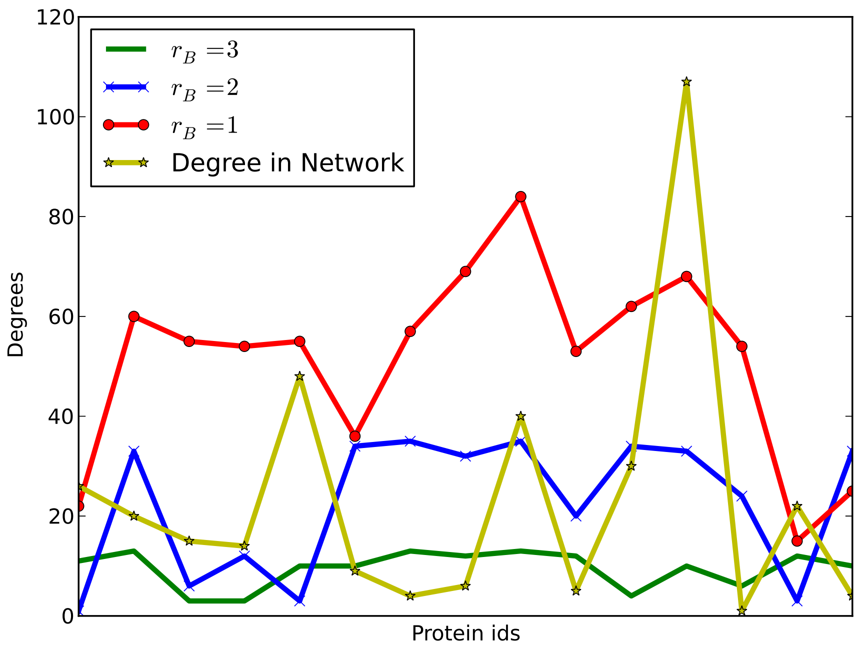

Figure S6: How the degrees vary for different radii and the actual degree in the network in MLP.

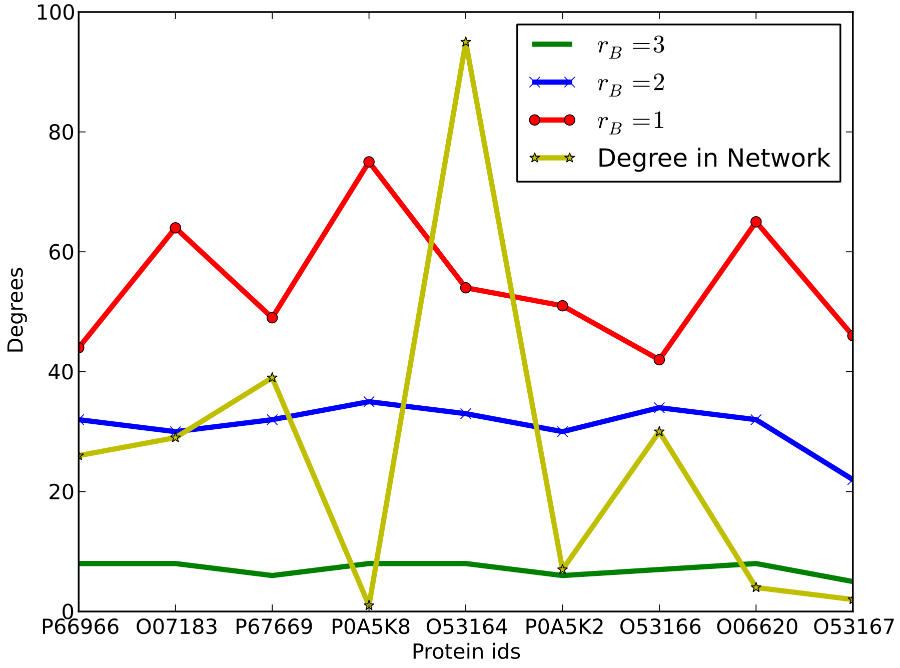

Figure S7: How the degrees vary for different radii and the actual degree in the network in MTB.
